# Examining the Extent of Contamination, Antibiotic Resistance, and Genetic Diversity of *Clostridioides* (*Clostridium) difficile* Strains in Meat and Feces of Some Native Birds of Iran

**DOI:** 10.1155/2023/3524091

**Published:** 2023-04-17

**Authors:** Akbar Ansarian Barezi, Amir Shakerian, Ebrahim Rahimi, Zahra Esfandiari

**Affiliations:** ^1^Department of Food Hygiene, Shahrekord Branch, Islamic Azad University, Shahrekord, Iran; ^2^Research Center of Nutrition and Organic Products (R.C.N.O.P), Shahrekord Branch, Islamic Azad University, Shahrekord, Iran; ^3^Nutrition and Food Security Research Center, Department of Food Science and Technology, School of Nutrition and Food Science, Isfahan University of Medical Sciences, Isfahan, Iran

## Abstract

*Clostridioides* (*Clostridium) difficile* (*C. difficile*) is one of the essential enteropathogens in humans and livestock and is a severe health threat, according to the Centre for Disease Control and Prevention. Also, antimicrobials are one of the most critical risk factors for *C. difficile* infection (CDI). The present study examined the infection, antibiotic resistance, and genetic diversity of the *C. difficile* strains in the meat and feces of some native birds (chicken, duck, quail, and partridge) in the Shahrekord region, Iran, from July 2018 to July 2019. Samples were grown on CDMN agar after an enrichment step. To determine the toxin profile, the *tcdA*, *tcdB*, *tcdC*, *cdtA*, and *cdtB* genes were detected via multiplex PCR. The antibiotic susceptibility of these isolates was examined using the disk diffusion method and followed based on MIC and epsilometric test. 300 meat samples of chicken, duck, partridge, and quail and 1100 samples of bird feces were collected from six traditional farms in Shahrekord, Iran. Thirty-five meat samples (11.6%) and 191 fecal samples (17.36%) contained *C. difficile*. Moreover, five toxigenic samples isolated had 5, 1, and 3 *tcdA/B*, *tcdC*, and *cdtA/B* genes. Out of the studied strains isolated from the 226 samples, two isolates belonging to ribotype RT027 and one isolated RT078 profile related to native chicken feces were observed from chicken sample. The antimicrobial susceptibility testing showed that all the strains are resistant to ampicillin, 28.57% are resistant to metronidazole, and 100% were susceptible to vancomycin. Based on the results, it can be concluded that the raw meat of birds might be a source of resistant *C. difficile* that poses a hygienic threat to the consumption of native bird meat. Nevertheless, further studies are essential to understand additional epidemiological features of *C. difficile* in bird meat.

## 1. Introduction

*Clostridioides difficile* (*Clostridium difficile*) is an anaerobic spore-forming bacterium that causes acute enteritis, colitis, and mortality particulary in susceptible people [1–3]. In 1978, this bacterium was the leading cause of antibiotic-induced diarrhea, called “antibiotic-associated diarrhea.” It is also responsible for pseudomembranous colitis and patient mortality, especially in the elderly [4]. Two major toxins, A and B, are responsible for the disease. A third toxin (binary toxin) being of uncertain clinical signifiance might be encountered in several “hypervirulent” strains such as ribotype 027 (RT027) or RT078 [4].

*C. difficile* was an essential nosocomial pathogenic bacterium, with healthcare facility environments considered the most important sites of infection. Since 2003, the severity and mortality rate of “*C. difficile* nosocomial infection” has increased significantly in North America and many European countries. Furthermore, several changes in bacterial epidemiology have been observed, including “community-acquired *C. difficile* infection,” the occurrence of the disease in young people without risk factors, emergence of highly invasive strains, the emergence of fluoroquinolone-resistant strains, increase in disease incidence, mortality, and similarities between *C. difficile* isolated from humans and animal feces [5, 6].

Currently, *C. difficile* infection causes 250,000 hospitalizations and 14,000 deaths per year [7, 8]. The confirmation of the presence of bacteria from the feces of animals that humans consumed meat attracted many researchers to study *C. difficile* in animal meat. Based on the multiple studies on this bacteria, it was introduced as an emerging pathogen in animals used as human food [9]. Due to the use of antibiotics in animals, food can be one of the main tools for transmitting antibiotic resistance genes from animal meat to humans. Fluoroquinolone antibiotics (such as ciprofloxacin) and tetracycline are widely used in the livestock diet and are even used to treat diseases that can cause antibiotic residues in animal meat [10]. Therefore, when humans consume animal meat, this antibiotic enters the body and can indirectly predispose a person to “nosocomial infection of *C. difficile*” [11]. As a result, most studies on *C. difficile* in various food sources are dedicated to meat and meat products. For instance, a study by Heise et al. on *C. difficile* in meat indicated the presence of *C. difficile* [12]. In poultry feces, a high proportion of toxigenic *C. difficile* was described in two studies from Zimbabwe. However, the highest prevalence recorded was found in a layer farm in Slovenia (62.3%) with a high genotypic diversity of the isolates, most of them nontoxigenic [13]. High genetic diversity but low prevalence in poultry was observed in India, Austria, and the Netherlands [13]. Studies showed that *C. difficile* spores could survive at 71°C in the minimum recommended time for cooking meat [13]. It is noteworthy that food sources, especially poultry, are a critical means of transmitting pathogenic bacteria [13]. Therefore, poultry became the subject of focus in the present study. Available data from the Middle East and the Far East, including 12 studies, showed the prevalence rates of toxin genes carrying *C. difficile* in meat samples ranged from 0% to 10.8%. Still, none of these studies investigated meat from different kinds of native birds [13]. The frequent isolation of ribotypes which are also found in humans constitutes a substantial overlap and makes poultry meat a potential source for *C. difficile* infection in humans [14]. Also, an earlier report in Zimbabwe reported the incidence of *C. difficile* in poultry feces, with a prevalence of 29% in rural habitats and 17.4% in broilers [14]. Therefore, the present study is aimed at evaluating the extent of contamination, antibiotic resistance, and genetic diversity of the *C. difficile* strains in some native birds such as chicken, duck, quail, and partridge in Iran.

## 2. Materials and Methods

### 2.1. Study Procedures

This study was carried out in Shahrekord region, Iran. To do this, 300 samples of chicken, duck, quail, and partridge meat and 1100 samples of their feces were collected by the random sampling method from 6 traditional local farms in Shahrekord from July 2018 to July 2019. The samples were transported on ice to the Research Center for Nutrition and Organic Products, Islamic Azad University, Shahrekord branch, Iran.

### 2.2. Microbiological Analysis

To isolate *C. difficile*, 5 grams of meat and feces samples of native birds were enriched in 45 mL of *C. difficile* broth (CDB) and were anaerobically incubated at 37°C for 10-15 days. The samples were cultured on C. *diffiicle* Moxalactam–Norfloxacin (CDMN) Agar. The phenotypic experiments identified multiple colonies from each sample, including colony morphology, gram staining, colony odor, and L-proline aminopeptidase disk. E.Z.N.A.® Stool DNA Kit extracted the DNA of colonies identified by the classical method.

### 2.3. Molecular Analysis

Multiplex PCR was used to detect the *tcdA*, *tcdB*, *tcdC*, *cdtA*, and *cdtB* genes of toxigenic *C. difficile* isolates. Briefly, the PCR mixture consisted of 2.5 *μ*L of PCR buffer, 2 *μ*L of each deoxynucleotide triphosphates (dNTP) at a concentration of 10 mM, 1 unit of single DNA polymerase enzyme, 5 *μ*L template DNA, and 0.1 *μ*L of each primer including *tcdA*, *tcdB*, *tcdC*, *cdtA*, and *cdtB* and sterilized deionized water. The thermal cycle involved the following steps: “initial denaturation” at 94°C for one minute, annealing at 94°C for 45 seconds, annealing at 52°C for 60 seconds, and extension at 72°C for 80 seconds, based on the method introduced by Lemee et al. [15].

The PCR products were visualized by electrophoresis on 1.5% agarose gel for 1 hour at 80 V. The gel was stained with ethidium bromide solution and isolated bands were observed using UV-doc [15].

PCR ribotyping was performed using 200 *μ*M of each dNTP mixture, 1.5 mM MgCl2, 2.5 U of Taq DNA polymerase, 50 *μ*L of each primer, 10 mM Tris-HCl (pH 8.8), 50 mM KCl, and 10 *μ*L of DNA extract. The total reaction volume was 100 *μ*L. The amplification was programmed for 30 cycles consisting of 95°C for 6 minutes in initial denaturation, 92°C for 60 seconds in denaturation, 55°C for 60 seconds in annealing, and 72°C for 6 minutes in extension steps. Amplicon product was loaded on 1.5% agarose gel for 6 hours at 80 V. Scanning by UV light was done after staining with ethidium bromide [16]. In the molecular tests, the strains of *C. difficile* ribotypes 027 and 078 were received from the Department of Pathobiology, University of Guelph, Canada, were used as positive controls.

### 2.4. Antibiotic Resistance Analysis

Antimicrobial susceptibility testing for different antibiotics was performed using gradient Etest (bioMérieux) and disc diffusion (Kirby Bauer). The minimum inhibitory concentration (MIC) was determined according to Clinical and Laboratory Standards Institute (CLSI) guidelines. Interpretive breakpoints for vancomycin were based on the European Committee for Antimicrobial Susceptibility Testing (EUCAST). The inhibition zone's diameter was interpreted based on the CLSI guidelines for the disc diffusion method. This method was used for resistance testing towards amoxicillin, ampicillin, ceftaroline, clindamycin, linezolid, meropenem, metronidazole, moxifloxacin, penicillin, pyracylene, tetracycline, and vancomycin on Mueller-Hinton agar medium according to the relevant protocols [17]. The diameter of the inhibition zone was read and interpreted after 48 hours of anaerobic incubation at 37°C. For antibiotic susceptibility testing, the innoculum was prepared at the 0.5 McFarland scale with a 24-hour young colony. These discs include amoxicillin (10 *μ*g), ampicillin (25 *μ*g), ceftaroline (64 *μ*g), clindamycin (16 *μ*g), linezolid (10 *μ*g), meropenem (25 *μ*g), metronidazole (8 g*μ*), moxifloxacin (10 *μ*g), penicillin (10 *μ*g), pyracylene (16 *μ*g), tetracycline (30 *μ*g), and vancomycin (4 *μ*g). Based on the specification of the disks, the antibiogram test report for each antibiotic was characterized as susceptible, resistant, and intermediate.

## 3. Results

The present study observed that 35/300 bird meat samples (11.6%) and 191/1100 fecal samples (17.3%) contained *C. difficile* based on morphological examination of the obtained colonies. White-gray, opaque, circular, and slightly raised colonies indicated the presence of *C. difficile* according the morphological examination technique. Furthermore, multiplex PCR results revealed that 10 samples from chicken (06), duck (03), and quail (01) had tcdA/B gene, one sample from chicken had *tcdC* gene, and 6 samples from chiken (04) and duck (02) had *cdtA/B* genes. Among them, respectively, 2 and 1 ribotype profiles of 027 and 078 were observed relating to native chicken feces (Table 1).

The interpretation of antibiotic resistance was based on CLSI and EUCAST guideline. Based on the obtained antibiogram results (following EUCAST/CLSI breakpoints), the highest resistance was related to ampicillin, and the highest susceptibility was related to metronidazole and vancomycin (Table 2).

## 4. Discussion

The present study assessed the degree of contamination, antibiotic resistance, and genetic diversity of Clostridioides (*C. difficile*) strains in meat and feces of some indigenous birds in Shahrekord region of Iran.

From this study, the overall prevalence of *C. difficile* in bird meat samples was 11.6% and 17.3% in feces samples. These results indicate that the meat and feces of some birds collected in this study are contaminated with *C. difficile*. Different studies have been conducted on the prevalence of *C. difficile* infection in poultry [12, 14, 18–21]. A study conducted in Isfahan and Khuzestan regions showed that the prevalence of *C. difficile* in beef, cow, sheep, goat, camel, and buffalo meat was 1.3% and 2.3%, respectively [22]. In relation to the present study, it can be inferred that the prevalence of *C. difficile* in meat and animal fecal samples in Iran is a real public health problem for the population. It could be considered a threat to public health because this bacterium is able to form very resistant spores that can persist in the environment for long periods of time, facilitating its transmission. It is therefore considered an opportunistic pathogen for humans and some animal species. Methods to remove the spores are therefore necessary. For example, Heise et al. [12] showed that a wet treatment of 85°C for 20 minutes can reduce the strain-dependent spore load by at least ~4 log units and that temperatures above 85°C are necessary to completely eliminate individual *C. difficile* spores in an aqueous environment.

In Zimbabwe in 2006, a study showed that 16.6% of indigenous chicken samples and 14.28% of fecal samples were contaminated with *C. difficile* [14]. In the work of Zamani et al. [21] on quail feces and meat in Iran, it was found toxinogenic *C. difficile* strains in feces samples. However, limited attention should be given to prevalence comparisons due to differences in study design. The portage of *C. difficile* in birds and wild animals would also be related to the fact that the bacterium is ubiquitous in the environment and several animal species can be colonized by this bacterium, e.g., pets and food animals and wild animals. Thus, contaminated meat, raw vegetables, and water may play an important role as sources of human infection since studies have linked strains of *C. difficile* isolated from animals, birds, and food to those identified in humans [23]. In addition, in Slovenia, a prevalence of 62.3% (highest prevalence) has been reported in laying hens [24]. In India, a high genetic diversity among *C. difficile* strains was noted with a prevalence of 14% in poultry [25]. The prevalence of *C. difficile* in poultry is lower in Austria (2.5%) and the Netherlands (2.3%) than in the USA (8.5%) and Canada (9.2%) [26, 27]. The prevalence of *C. difficile* in Sweden and Austria is 2.7% [28]. According to studies by Weese et al. [18] and Guran and Ilhak [29], *C. difficile* was isolated from 12% and 8% of broiler meats, respectively. *C. difficile* was isolated from fecal samples in 60% of broilers on the poultry farm in Slovenia [24]. Similarly, Harvey et al. reported that *C. difficile* was isolated from 2% of chicken fecal samples and 12% of broiler meat samples [27]. It should be noted that the results of the present study are consistent with previous reports of chicken meat contamination, except that the fecal contamination rate in our study was higher.

In the present study, the *tcdA*, *tcdB*, *cdtA*, and *cdtB* genes were studied in chicken, duck, and quail because they are considered the most common genes for *C. difficile* toxin typing. According to Weese et al. [18], all strains isolated from broiler meat (12% of total samples) had genes encoding toxins A and B. *C. difficile* was isolated from 26/203 (12.8%) chicken samples; 10/111 (9%) thighs, 13/72 (18%) wings, and 3/20 (15%) legs and all isolates were ribotypes 078 [18]. Three strains of *C. difficile* were noted to possess the *tcdA* and *tcdB* genes, 2 for ribotype 027, and one for the 078-profile linked to native chicken feces (Table 1). The results show that less than one percent of the birds carry these ribotypes, and these data corroborate other previous observations [26, 30–32]. It is worth remembering that ribotypes 027 and 078 are the most virulent and the diarrheal outbreaks due to *C. difficile*. Ribotypes 027 and 078 are often associated with infectious diarrhea in Iranian hospitals [33]. Three toxigenic strains were identified in this study. These results shows that measures must be taken against the dissemination of virulent and toxigenic strains in order to avoid diarrheal epidemics linked to these strains. In addition, the frequent isolation of ribotypes constituted a significant overlap, making poultry meat a potential source of *C. difficile* infection in humans [32–35]. From these data, it could be inferred that consumption of broilers and contaminated chicken meat could be a source of human disease. The *tcdC* gene was found in only one strain. According to the literature, this gene is present in all toxigenic strains. This result is probably due to the technique used or the working conditions. But it also opens other research perspectives with advanced techniques to detect this gene in identified isolates, such as whole-genome sequencing.

Antimicrobial resistance of *C. difficile* is highly variable in different birds and countries [6]. The results of the antibiotic susceptibility study of isolated bacteria showed that *C. difficile* strains exhibited a high rate of resistance to ampicillin and susceptibility to metronidazole and vancomycin. Although the susceptibility of *C. difficile* strains is studied, there is a lack of information on resistance to fidaxomicin, meropenem, and piperacillin/tazobactam [36]. According to Saha et al. [37], *C. difficile* resistance to vancomycin is increasing. Metronidazole and vancomycin are antibiotics to treat *C. difficile* infections. Although resistance to metronidazole and vancomycin is not yet a major problem, the reduced sensitivity to these antibiotics has progressively increased, which underlines the need for constant monitoring and regulation of the use of these molecules. Low resistance to tetracycline, clindamycin, and moxifloxacin was noted in this study. These results are contrary to those of Heidari et al. [38] who obtained high level resistance of tetracycline, clindamycin, and moxifloxacin.

The limitation of the present study is the lack of accessibility to reference profiles of *C. difficile* for a complete comparison of the isolates found with the frequently used international profiles for poultry such as RT001, RT002, RT014, and RT020. This study is also limited by the fact that it covers only Shahrekord region, Iran.

## 5. Conclusion

The current study concluded that feces and meat of poultry and native birds, including chicken, duck, quail, and partridge, can be sources of pathogenesis through *C. difficile* in Iran. The consumption of this group of animals is the favorite of Iranian people. Therefore, appropriate cooking of these animals is recommended. More studies are suggested to understand the different aspects of the epidemiology of *C. difficile* in Iran.

## Figures and Tables

**Table 1:** Contamination and genetic diversity of *C. difficile* strains in chicken, duck, quail, and partridge.

Type of native bird	Number of meat/feces samples	Number of samples infected with *C. difficile* of meat/feces^∗^	Toxin gene profile				
			*tcdA*	*tcdB*	*tcdC*	*cdtA*	*cdtB*
Chicken	90/300	22/95	+3	+3	+1	+2	+2
Duck	60/250	12/88	+1	+2	—	+1	+1
Quail	60/250	1/5	+1	—	—	—	—
Partridge	90/300	0/3	—	—	—	—	—
Total	300/1100	35/191	5	5	1	3	3

^∗^Detection through L-proline aminopeptidase test for meat/feces.

**Table 2:** Examining the resistance and susceptibility of *C. difficile* strains in chicken, duck, partridge, and quail samples of current study.

Antibiotics	Range	Concentration of antibiotics	Resistance	Susceptible (percentage)	Intermediate (percentage)	Resistant (percentage)
Amoxicillin	1-10	7	Intermediate	0	18 (51.4)	24 (5.68)
Ampicillin	5-25	5	Resistant	0	0	35 (100)
Ceftaroline	6-64	58	Intermediate	5 (14.28)	18 (51.4)	12 (34.28)
Clindamycin	1-16	1	Resistant	0	11 (31.4)	24 (68.57)
Linezolid	1-10	6	Intermediate	11 (31.4)	6 (17.1)	18 (51/42)
Meropenem	5-25	19	Intermediate	11 (31.4)	12 (34.3)	12 (34.28)
Metronidazole	0.125-80	80	Susceptible	35 (100)	0	0 (0)
Moxifloxacin	1-10	5	Intermediate	12 (34.2)	12 (34.3)	10 (28.57)
Penicillin	1-10	6	Intermediate	0	30 (85.7)	5 (14.28)
Pyracylene		13	Intermediate	12 (34.2)	12 (34/3)	11 (31.42)
Tetracycline		22	Intermediate	11 (31.4)	14 (42.9)	9 (25.71)
Vancomycin	0.25-4	4	Susceptible	35 (100)	0	0

## Data Availability

All data generated or analyzed during this study are included in this article.

## Additional Points

*Novelty Impact Statement.* (i) *C. difficile* prevalence in meat samples was 11.6% and in fecal samples was 31.6%. (ii) The highest susceptibility for *C. difficile* isolates was related to metronidazole.
